# Parallel perfusion imaging processing using GPGPU

**DOI:** 10.1016/j.cmpb.2012.06.004

**Published:** 2012-12

**Authors:** Fan Zhu, David Rodriguez Gonzalez, Trevor Carpenter, Malcolm Atkinson, Joanna Wardlaw

**Affiliations:** a Data-Intensive Research Group, School of Informatics, University of Edinburgh, Edinburgh, UK; b SFC Brain Imaging Research Centre, Division of Clinical Neuroscience, University of Edinburgh, Edinburgh, UK

**Keywords:** Local AIF, Perfusion imaging, Deconvolution, Parallelization, GPGPU

## Abstract

**Background and purpose:**

The objective of brain perfusion quantification is to generate parametric maps of relevant hemodynamic quantities such as cerebral blood flow (CBF), cerebral blood volume (CBV) and mean transit time (MTT) that can be used in diagnosis of acute stroke. These calculations involve deconvolution operations that can be very computationally expensive when using local Arterial Input Functions (AIF). As time is vitally important in the case of acute stroke, reducing the analysis time will reduce the number of brain cells damaged and increase the potential for recovery.

**Methods:**

GPUs originated as graphics generation dedicated co-processors, but modern GPUs have evolved to become a more general processor capable of executing scientific computations. It provides a highly parallel computing environment due to its large number of computing cores and constitutes an affordable high performance computing method. In this paper, we will present the implementation of a deconvolution algorithm for brain perfusion quantification on GPGPU (General Purpose Graphics Processor Units) using the CUDA programming model. We present the serial and parallel implementations of such algorithms and the evaluation of the performance gains using GPUs.

**Results:**

Our method has gained a 5.56 and 3.75 speedup for CT and MR images respectively.

**Conclusions:**

It seems that using GPGPU is a desirable approach in perfusion imaging analysis, which does not harm the quality of cerebral hemodynamic maps but delivers results faster than the traditional computation.

## 1 Introduction

With the development of computed tomography (CT) [1,2] and magnetic resonance (MR) imaging [3,4], perfusion imaging becomes a very powerful clinical tool for evaluation of brain physiology. They can be used to evaluate brain function via assessment of cerebral perfusion parameters.

The main applications of brain perfusion imaging are acute stroke and brain tumors. In the case of acute stroke, the information obtained from brain perfusion imaging can be used to evaluate the appropriateness of administering thrombolytic treatment, which can help to reduce the final volume of dead tissue, but has some risks such as hemorrhages. The results are used to evaluate the possible benefits. In the case of tumors, they are used to distinguish tumor characteristics and follow tumor development, possibly also after treatment to see whether it has been effective.

Evaluating tissue time–concentration curve of a contrast agent intensity after its injection, has become possible on time scales comparable with the mean transit time (MTT). To achieve this, deconvolution is used in perfusion imaging to obtain the Impulse Response Function (IRF) that is then used to create parametric maps of relevant hemodynamic quantities such as cerebral blood flow (CBF), cerebral blood volume (CBV) and mean transmit time [5–7]. Cerebral blood flow indicates the volume of blood flowing through a given voxel in a given time. Cerebral blood volume refers to the volume of blood in a given voxel of brain tissue. Mean transit time designates the average time blood takes to flow through a given voxel of brain tissue, it is commonly measured in seconds. Time To Peak (TTP) and Time of Arrival (TA) are two other parameters often be measured [8]. TA refers to the time of arrival of the contrast agent in the voxel after injecting contrast agent. TTP refers to corresponding time of the maximum contrast concentration. In previous studies, singular value decomposition (SVD) and its variants were proved to be applicable to perform deconvolution in perfusion imaging [9]. As the raw data obtained from CT or MR scanners is not noise free and as deconvolution is very sensitive to noise, truncated SVD is used to minimize the noise impact [10–13].

In clinical practice, a global AIF for the entire brain can be determined from voxels near a major artery feeding the brain. However, the global AIF technique is based on the assumption that the contrast agent reaches every voxel of the brain at the same time. In the case of acute stroke, the contrast arrival time can be different and the assumption is not satisfied. As a result, using a global AIF for the entire brain is not very accurate [14,15].

The other solution is to use local AIFs [16–19]. Instead of using a global AIF generated from voxels near the major artery for whole brain, different local AIFs are used for a single scan. Each local AIF is generated by measuring a small set of blood vessels in a specified area near the voxel of interest. Lorenz et al. [16] had shown that localized AIFs are feasible and provide more useful perfusion results.

However, using local AIFs leads to fairly slow performance, in the worst case, the perfusion-imaging analysis takes more than half an hour compared with the running time of global AIFs based methods which is a couple of minutes. According to Saver's experiment in 2006 [20], during 30 min, 57.6 million neurons die. In the same minutes, your brain loses 41.4 billion synapses and 360 km of axonal fibers. Since a stroke is a medical emergency and every second counts, the sooner results are delivered in diagnosis, the less damage will be caused to a patient's brain. Obviously, half an hour is not a reasonable option for clinical diagnosis. Therefore, a parallel implementation of perfusion-imaging analysis which brings performance speedup without quality lose is very promising to help using local AIFs in perfusion imaging.

In this paper, we present a GPGPU-based brain perfusion imaging analysis implementation using the CUDA programming model. We also compared the performance of the serial and parallel perfusion imaging analysis methods.

## 2 Background and methods

### 2.1 Perfusion imaging algorithm

Ostergarrd et al. [11,12] and Wurestan et al. [13] have shown that an accurate CBF can be determinated using deconvolution of a tissue time–concentration curve and an AIF. From a CT or MR scanner, we get a series of brain images at different sampling times. For each voxel, we collect data at specific time intervals to build a tissue time–concentration curve of contrast agent intensity, which is also called volume of fluid (VOF) curve. This curve will be referred to as *C*_*t*_.

A local AIF matrix is created from the local AIF vector as follows:

(1) $$C_{a}=\Delta t\left(\begin{array}{cccc} C_{a}(t_{1}) & 0 & \cdots & 0\\ C_{a}(t_{2}) & C_{a}(t_{1}) & \cdots & 0\\ \vdots & \vdots & \ddots & \vdots \\ C_{a}(t_{N}) & C_{a}(t_{N-1}) & \cdots & C_{a}(t_{1}) \end{array}\right)$$

where (*t*_1_, *t*_2_, …, *t*_*N*_) is the sampling time, (*Ca*(*t*_1_), *Ca*(*t*_2_), …, *Ca*(*t*_*N*_)) is an arterial input function given as an input and Δ*t* is time scale.

In perfusion imaging, the output we want to obtain is Impulse Response Function (IRF), which is referred to as *h*.

The volume of fluid, *C*_*t*_, the *C*_*a*_, and IRF *h* satisfies the following equation:

(2) $$C_{t}=C_{a}⊗h+\epsilon$$

where ⊗ denotes convolution and *ϵ* is the noise.

Finally, the CBF, CBV and MTT for each voxel are calculated as follows:

(3) CBF=Max(h)

(4) $$\mathrm{CBV}=\int_{0}^{\infty }h(t)\;\mathrm{dt}$$

(5) $$\mathrm{MTT}=\frac{\mathrm{CBF}}{\mathrm{CBV}}$$

*Singular value decomposition* (SVD) is one of the most popular techniques to solve deconvolution problems in perfusion imaging. Suppose *C*_*a*_ from Eq. (1) is an *m*-by-*m* matrix, there exists a factorization such that:

(6) $$C_{a}=U\cdot W\cdot V^{T}$$

where *U* is an *m* × *m* unitary matrix, *W* is *m* × *n* diagonal matrix and V^*^ is the transpose of an *n* × *n* unitary matrix *V*. A common convention is to order the diagonal matrix *W* in a decreasing order and these diagonal entries of *W* are known as the singular values of original matrix *C*_*a*_.

The $C_{a}^{-1}$ can then be written as:

(7) $$C_{a}^{-1}=V\cdot W^{-1}\cdot (U^{T})$$

To solve the deconvolution problem in Eq. (2), the solution can be simply delivered after applying SVD:

(8) $$h=V\cdot W^{-1}\cdot (U^{T}\cdot C_{t})$$

Furthermore, as rows in *C*_*a*_ in Eq. (2) are close to linear combinations, the deconvolution is an ill-posed problem, hence, it is very sensitive to noise. Truncated SVD is introduced to minimize the effect of noise. In truncated SVD, a threshold is added and elements of the diagonal matrix *W* whose value is smaller than this threshold will be set to zero [11,12].

### 2.2 CUDA for GPGPU

GPUs are especially well suited to address data parallel computation problems that the same program is executed on a large number of data elements in parallel especially for those tasks which can be split into single instruction, multiple data (SIMD) subtasks. For example, GPUs are good at handling matrix operations since the same transformation is executed on every element within the matrix and there is almost no dependence between different elements.

Compute Unified Device Architecture (CUDA) is a parallel computing architecture developed by NVIDIA in 2006 [21] with an associated software toolkit. It is the entry point for developers who prefer high-level computer programming, compared with Open Computing Language (OpenCL), which is the entry point for developers who want low-level Application Programming Interfaces (APIs). The CUDA programming architecture is very well suited to expose the parallel capabilities of GPUs.

C for CUDA offers programmers a simple way to write C-like programs for GPGPUs. It consists of a set of extensions to the C language for code running on CPUs and a runtime library for code running on GPUs. It significantly reduces the runtime overhead of GPGPU applications. As a result, CUDA has become one of the most popular programming languages for GPU programming. In addition, although it access GPUs via high-level APIs compared to other architecture such as OpenCL, it also allows programmers to access use low-level APIs to avoid the overhead common with graphics APIs.

#### 2.2.1 CUDA data and control flow

**Fig. 1:**
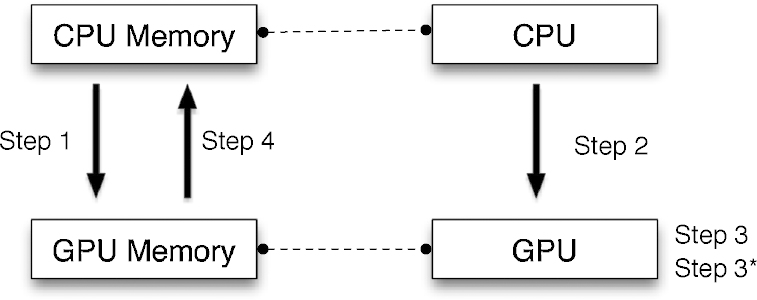
CUDA data and control flow. This figure indicates the four steps of a CUDA data and control flow.

Fig. 1 is a typical example of GPU function execution in CUDA:

1. Copy data from main memory to GPU memory.
2. CPU instructs the GPU to start processing.
3. GPU executes in parallel on each core.
3.^*^ Wait for completion. This step happens the same time as step 3.
4. Copy the result from GPU memory to main memory.
5. CPU acts on result, and may return to step 1 in order to execute another GPU function.

## 3 Algorithm

Truncated singular value decomposition mentioned above is used to calculate the IRF. The following defines the variables in the pseudo code.

**Table:** 

*Input*: 4D MR or CT image data stored in Nifti format [22] file.
*Output*: A set of CBF, CBV and MTT colored maps.
*Time*: the number of time intervals.
*Dim1, Dim2, Dim3*: the size of each dimension.
*Size*: the size of each 3D brain image which equals to *Dim*1 × *Dim*2 × *Dim*3.
*A()*: a 4D array used to store data directly read from brain images.
*A*′*()*: a 4D array used to store data after reorganization.
*A*″*()*: a 4D array used to store data after denoising.
*IRF*: a 1D array used to temporary store the result of deconvolution.
*CBF(),CBV(),MTT()*: 3D arrays used to store the analyzed result.
*CPU.A*: Parameter A is stored on the CPU.
*GPU.A*: Parameter A is stored on the GPU.
*GPU A* ← *B*: Operation *A* ← *B* is executed on the GPU.
*GPU.A* ← *CPU.A*: Copy data from CPU to GPU.
*CPU.A* ← *GPU.A*: Copy data from GPU to CPU.

### 3.1 Using GPGPU to decomposite a matrix

GPGPUs can be used in matrix decomposition problems [23,24]. Lahabar et al. [24] compared the performance in terms of speed of SVD in MATLAB, SVD in Intel Math Kernel Library (MKL) 10.0.4 LAPACK and his implementation on GPU using CUDA. Their test environment was an *Intel Dual Core 2.66 GHz* PC and *NVIDIA GTX 280* graphics processor. Their study focuses on evaluating the performance of parallel and serial versions of the SVD algorithms rather than some specific application of SVD. In their method, they divided the decomposition into small tasks so that each GPU thread will only handle the calculation corresponding to one element of the matrix a time.

As the largest data set in our case is a 80 × 80 matrix, using GPGPU to split the matrix decomposition is not suitable according to the results in Table 1 from [24]. From this table, SVD using GPU will improve the performance only if the matrices are larger than 1K × 1K but will impair the performance for smaller matrices. In our case, the matrices we want to decompose range from 44 × 44 to 80 × 80 which are too small to obtain improvement. As a result, using GPGPU for individual matrix decomposition will not show performance improvement in our case. Therefore, the deconvolution task is not parallelized using lower level parallelism.

Algorithm 1 Serial perfusion imaging analysis

**Table:** 

1	*A*(1 : *Time*, 1 : *Size*)← 4D MR or CT image data
2	**if** DoImageDenoising = *true*
3	**then***A*′(1 : *Size*, 1 : *Time*)← reorganise *A*(1 : *Time*, 1 : *Size*)
4	**else***A*″(1 : *Size*, 1 : *Time*)← Denoise and reorganise *A*′(1 : *Size*, 1 : *Time*)
5	**for***i* ← 1 **to***dim*
6	**do** Generate *localAIF*(1 : *Time*)
7	*IRF*(1 : *Time*)← Deconvolution result (A”(i,1:Time) &localAIF(1:Time))
8	*CBF*(*i*) ← *Max*(*IRF*(1 : *Time*))
9	*CBV*(*i*) ← *Sum*(*IRF*(1 : *Time*))
10	*MTT*(*i*) ← *CBV*(*i*)/*CBF*(*i*)
11	CBF colored map ← CBF(1:Size)
12	CBV colored map ← CBV(1:Size)
13	MTT colored map ← MTT(1:Size)

### 3.2 Serial perfusion imaging analysis

The algorithm for perfusion-imaging analysis without parallelization can then be written as Algorithm 1.

**Source image loading**: The first step (Line 1) is to load MR or CT imaging data stored in neuroimaging informatics technology initiative (NIfTI) format file. The computational complexity of step one is *O*(*time* × *Dim*1 × *Dim*2 × *Dim*3).

**Fig. 2:**
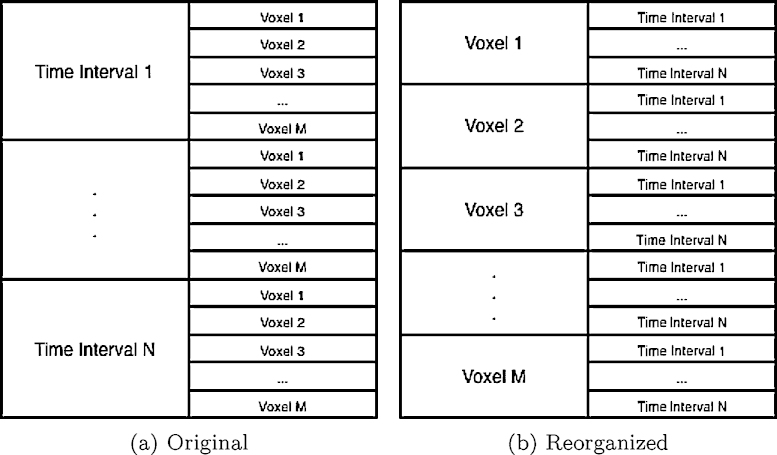
Data structure. (a) How data are structured in the source file. (b) The data structure to which it is transformed to maximize localization.

**Data reorganization**: For each voxel, to generate tissue time-concentration curves in deconvoluting step (Line 3) requires data from all of the time intervals. However, images are originally stored in another way where all of the voxels at a given time interval are grouped together (Fig. 2(a) corresponding to the order in which data arrives from a scanner. This organization of data will dramatically increase cache swap overhead. So the second step is to reorganize data from the form of [*time*][*Dim*3][*Dim*2][*Dim*1] into the form of [*Dim*3][*Dim*2][*Dim*1][*time*] (Fig. 2b) to maximal data localization. The computational complexity of this step is *O*(*time* × *Dim*1 × *Dim*2 × *Dim*3).

**Denoising (optional)**: As blood always flows from one cell to its neighbors, the intensity values should be continuous. This allow us to use an image-level denoising method (Line 4) such as applying 2D, 3D and 4D weighted mean filters. The computational complexity of this step is also *O*(*time* × *Dim*1 × *Dim*2 × *Dim*3).

**Deconvolution**: Lines 6–10 perform the deconvolution. This operation runs voxel by voxel. The most expensive part in the deconvolution is to decompose local AIF matrices, a *time*^2^ matrix composed from given AIF vector to solve deconvolution problem [11], using singular value decomposition whose computational complexity is *O*(*time*^3^) for each AIF matrix according to our implementation. The computational complexity of deconvolution can be roughly considered as the same as decomposition: *O*(*time*^3^).

Furthermore, as voxel-based deconvolution in Line 5–10 needs to be repeated *Dim*1 × *Dim*2 × *Dim*3 times, the overall computational complexity is *O*(*Dim*1 × *Dim*2 × *Dim*3 × *time*^3^). This is the most expensive part of the whole workflow, more details can also be found in Section 4.2.

**Result generation**: The last step (Lines 11–13) is to write parametric maps using the results generated from deconvolution. The computational complexity of this step is *O*(*Dim*1 × *Dim*2 × *Dim*3).

**Overall**: The steps *source imaging loading, data reorganization, denoising* and *result generation* can be assumed to be small compared to the *deconvolution* step provided that *time* > 2. This assumption is always true in perfusion imaging where time is on the order of 10^1^ − 10^2^. Hence, the overall computational complexity for perfusion-imaging analysis is *O*(2 × *Dim*1 × *Dim*2 × *Dim*3 × *time* + *Dim*1 × *Dim*2 × *Dim*3 × *time*^3^) which can be considered as *O*(*Dim*1 × *Dim*2 × *Dim*3 × *time*^3^) which is the same as the computational complexity of deconvolution step.

Algorithm 2 Parallel perfusion imaging analysis

**Table:** 

1	*CPU* . *A*(1 : *Time*, 1 : *Size*)← 4D MR or CT image data
2	*GPU* . *A*(1 : *Time*, 1 : *Size*) ← *CPU* . *A*(1 : *Time*, 1 : *Size*)
3	GPU: Parallel do, shared(*A*, *A*′, *A*″)
4	**if** DoImageDenoising = *true*
5	**then***GPU* . *A*′(1 : *Size*, 1 : *Time*)← reorganise *GPU* . *A*(1 : *Time*, 1 : *Size*)
6	*GPU* . *A*″(1 : *Size*, 1 : *Time*) = *GPU* . *A*′(1 : *Size*, 1 : *Time*)
7	**else***GPU* . *A*″(1 : *Size*, 1 : *Time*)← Denoise and reorganise *GPU* . *A*(1 : *Time*, 1 : *Size*)
8	GPU: Parallel do, *private*(*localAIF*, *i*, *IRF*), *shared*(*A*, *CBF*, *CBV*, *MTT*)
9	**for***n* ← 1 **to***Dim*3
10	**do for***i* ← 1 **to***Dim*1 × *Dim*2
11	**do** Generate *localAIF*(1 : *Time*)
12	*IRF*← Deconvolution result (GPU.A”(i+n × Dim1 × Dim2,1:Time) &localAIF(1:Time))
13	*GPU* . *CBF*(*i* + *n* × *Dim*1 × *Dim*2) ← *Max*(*IRF*)
14	*GPU* . *CBV*(*i* + *n* × *Dim*1 × *Dim*2) ← *Sum*(*IRF*)
15	*GPU* . *MTT*(*i* + *n* × *Dim*1 × *Dim*2) ← *GPU* . *CBV*/*GPU* . *CBF*
16	*CPU* . *CBF*(*slice n*) ← *GPU* . *CBF*(*slice n*)
17	*CPU* . *CBV*(*slice n*) ← *GPU* . *CBV*(*slice n*)
18	*CPU* . *MTT*(*slice n*) ← *GPU* . *MTT*(*slice n*)
19	CBF colored map ←*CPU* . *CBF*(*slice n*)
20	CBV colored map ←*CPU* . *CBV*(*slice n*)
21	MTT colored map ←*CPU* . *MTT*(*slice n*)

### 3.3 Parallel perfusion imaging analysis

As GPGPUs is an ideal solution for matrix operations; it can be expected to improve the performance of *Data reorganization* and *Denoising* steps. In the deconvolution step, the deconvolution of different voxels are ideally parallel tasks, so that there is little effort required to separate the problem into parallel tasks and there is no dependency or communication between those parallel tasks, parallel implementation can be easily achieved. The parallel algorithm for the whole workflow can then be written as Algorithm 2.

**Source image loading**: For the serial algorithm, the first step in the parallel implementation is to load images into CPU memory (Line 1). The implementation of this step is exactly the same as before, so its computational complexity remains *O*(*time* × *Dim*1 × *Dim*2 × *Dim*3).

**GPU memory copy in**: Line 2 is an extra step, as mentioned in Section 2.2.1, data which will be used in the following step will be copied from CPU memory to GPU memory. The computational complexity of this step is *O*(*time* × *Dim*1 × *Dim*2 × *Dim*3).

**Reorganization and denoising** The *Data reorganization* (Line 5) and *Denoising* (Line 7) steps can be considered as matrix transformations, which CUDA is good at handling, with 4D input array. Each GPU thread is responsible for one element in the input array. To put it differently, each GPU thread is in charge of one and only one element which presents the intensity value of one voxel at one time interval. The thread read the intensity value and then stores it to right place in reconstructed array. The task for each thread is light and the number of threads is production of the number of voxels and number of time intervals.

**Fig. 3:**
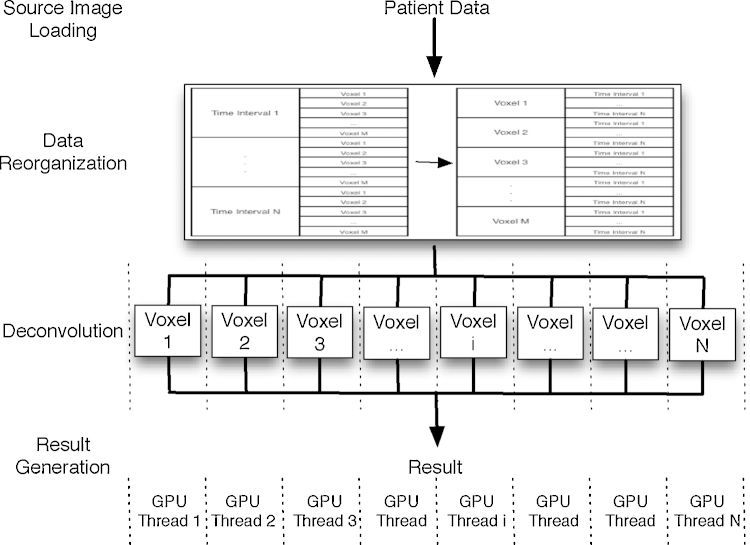
GPGPU parallelization workflow.

**Deconvolution**: Lines 7–15 are the most expensive part of the whole workflow. The main part of deconvolution, decomposition of each local AIF matrix, whose size is 80 × 80, is not large enough to be parallelized (Section 3.1). Consequently, we simply assign each decomposition to a different GPU thread. As illustrated in Fig. 3, each GPU thread corresponds to the deconvolution of one pixel. Therefore, hundreds of voxel deconvolutions can be performed concurrently.

**GPU memory copy out**: Lines 16–18 is another extra step from serial version. In this step, results of deconvolution will be copied back from GPU memory to CPU memory. The computational complexity of this step is *O*(*Dim*1 × *Dim*2 × *Dim*3).

**Result generation**: The last step, *Drawing parametric maps*, is also the same as in the serial version. The computational complexity is also *O*(*time* × *Dim*1 × *Dim*2 × *Dim*3).

**Overall**: The parametric maps produced by serial and parallel implementations are identical. In other words, the quality of the results is not compromised. The computational complexity of the parallel implementation is *O*(*Dim*1 × *Dim*2 × *Dim*3 × *time*^3^), which is the same as the computational complexity of serial implementation. However, in parallel programming, computational complexity is not the only factor affecting the performance. Performance is highly related to how well the code is parallelized.

### 3.4 Space complexity for deconvolution

In principle, the amount of data transferred between the CPU memory and GPU memory should be kept as low as possible. Intermediate data structures should be created in GPU memory and freed after use without being copied to CPU memory.

Using SVD, three *time*^2^ local arrays and one *time* array are required for each voxel to store the input and output matrices. Furthermore, four *time*^2^ arrays are required when calculating the inverse matrix in SVD. The memory of the input matrix can be re-used in inverse matrix calculation and the output matrices re-use the memory that was allocated to calculating the inverse matrix. So the space complexity is (*time*^2^) for each voxel and (*time*^2^× number of voxels) for each scan. Due to the large number of voxels, the whole process requires a large amount of memory.

Taking a typical MR image size (dim1 × dim2 × dim3 × number of time intervals) to be 128 × 128 × 22 × 80, with each intensity value stored in a *float* variable as an example, about 200 KB^1^ memory is required for each voxel. Unfortunately, that exceeds CUDA local memory limitation which is 16 KB per GPU function. As a result, these arrays have to be declared in global memory which leads to another problem that 80 GB^2^ memory is required if local arrays for the whole image are declared simultaneously. This exceeds the overall memory (4.0 GB) available on current GPUs. Considering that local arrays are temporarily used in the deconvolution within each voxel, its space can be reused by another voxel after its deconvolution is finished. Therefore, the solution to the memory problem is to declared a certain size of memory in global memory exclusively for local arrays to use, and assign the memory to one voxel's deconvolution and recycle it when that deconvolution is complete.

Choosing the size of memory is a compromise between memory management cost and memory usage. On the one hand, declaring more memory can enable more deconvolutions to execute at the same time but it requires a large amount of memory. That reduces the effort to manage local memory but dramatically reduces the overall memory available for rest of the processes. On the other hand, if the size of memory for local arrays to use is too small, some of the GPU cores have to be idle as they are not able to obtain memory to execute.

In our experiment, the size of local arrays’ memory has been set to 128× 128 × 4 ×80^2^ × *size of (float)* which means 3 GB memory is declared to cover arrays for 128 × 128 voxels. Memory management costs can be kept at a low level as memory only needs to be re-used fewer than thirty times during the analysis. Furthermore, it leaves enough memory for the rest of the analysis.

### 3.5 Memory bandwidth analysis

The size of input data is 55 MB^3^; for output, taking bitmap file format as an example, each voxel requires three *unsigned char* type of variables to store the RGB color information, it only costs about 1 MB^4^ for one type of hemodynamic quantity map.

As the peak memory bandwidth for GPUs exceeds 180 GB/s (since 2010), which is very fast compared to the memory bandwidth for CPUs (less than 40 GB/s), programs based on GPUs are less sensitive to data transfer rates than CPU programs. The GPU used in our experiment has a memory bandwidth of 102.4 GB/s. The cost of read/write memory can be kept as low as a few milliseconds, which only contributes a little to the overall running time. Therefore, this experiment does not make use of the shared memory to reduce memory bandwidth bottleneck.

### 3.6 Other parallel implementations used as comparison

Two other parallel approaches using OpenMP (shared memory parallel architecture) and MPI (message passing parallel architecture) are also implemented in the experiment. Similar to the implementation using GPGPU, these two approaches use upper-level parallelism which focuses on the deconvolution step. Unlike our GPGPU implementation, which assigns one voxel to one GPU thread, our OpenMP and MPI implementations divide all of the voxels in to several groups and assign one group to one CPU thread. This is because of the cost of CPU scheduling is heavier than it is for GPU's, this arrangement reduces that overhead.

### 3.7 GPU kernel program fragment

**Fig. 4:**
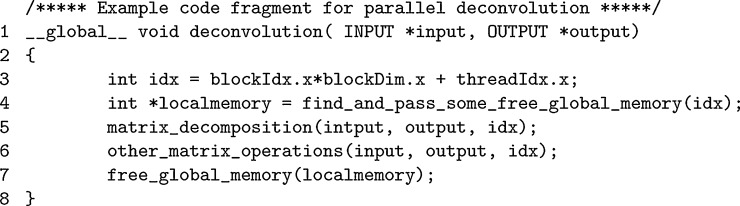
GPU kernel program fragment.

Fig. 4 shows a GPU kernel code fragment for the deconvolution step. The deconvolution task for each voxel is processed by one GPU thread. So the number of GPU threads equals to the number of voxels. Within each deconvolution, the task is much heavier than it is in the matrix transformation, there is a matrix decomposition and several other matrix operations. Different threads operate on different voxels and there is no data dependency between different threads. As a result, no synchronization is required in the GPU threads.

For the reason stated in Section 3.4, the first step in deconvolution is to request a chunk of memory from the pre-allocated global memory and use it as local memory, which only gets used inside the current thread. After that, matrix decomposition and other matrix operations can be processed in the same way as they are in the regular deconvolutions. The last step is to release the chunk of local memory back to the global pool for reuse.

## 4 Performance

### 4.1 Experimental environment

In our experiment, the worker node we use contains two *Intel*(*R*)*Xeon*(*R*) CPU cores and connects to a Tesla C1060 GPU which provide 240 GPU cores in total. The frequency of each CPU core is 3.0 GHz and the frequency of GPU core is 1.44 GHz. The overall CPUs memory is 8.0 GB and their cache size is 4 MB each. The GPU's single precision floating point performance (peak) is 933.12 GFLOPS and it has 2.0 GB of global memory and 8 KB of shared memory. CUDA 4.0.2.1221 by NVIDIA released in May 2011 has been used as the programming language. Furthermore, OpenMPI 1.4.2 has been used for MPI programming [26] and Intel compilers (version 11.0) has been used for OpenMP programming [27]. Furthermore, since there are only four cores in total, the number of threads are set to four in both of the OpenMP and MPI implementations.

One of the test data we used is simulated images each containing 128 × 128 × 22 voxels and the number of time intervals is 80, which is one of the size of MR images. Another test data in the experiment consists of 128 × 128 × 11 voxels with 44 time intervals, which is one of the size of CT images. Input data is stored using *short* data type, which requires 2 Bytes for each element. The results showed below are the arithmetic mean of ten repeated tests. There is almost no connection between the processing time and the features of the patients. The only factor that matters is the size of the images.

### 4.2 Performance for each step

Table 2 indicates our measurement of the performance for each step in the whole workflow.

The steps *Brain data load* and *Draw parametric maps* are not suitable for parallelization and their running time in parallel version can be considered as the same as in the serial version.

In parallel deconvolution, the first step of parallel workflow is to copy data from CPU memory to GPU memory. The input data is about 220 MB, which is mainly an array with 128 × 128 × 22 × 80 *short* elements. The copying takes 0.17 s. The result size to be moved back from GPU memory to CPU memory is much smaller and only takes 0.01 s to perform the copy back operation.

In serial deconvolution, the *Reorganization* & *Denoising* step, prior to deconvolution, takes 4.3 s compared to the 1.1 s for reorganization only. After applying parallelization to these steps, the performance dramatically increased to 0.01 s. The speedup factors are 430 and 110, respectively.

The running time of the *Deconvolution* step, the most expensive one, reduced from 2108 s to 564 s after applying parallelization. The speedup factor is 3.74.

This result supports the computational complexity analysis mentioned in Sections 3.2 and 3.3.

### 4.3 Overall performance

Table 3 shows the overall speedup improvement gained from GPGPU. As the running time is dominated by *Deconvolution* step, although other steps can be improved by large speedup factors, the overall running time can be roughly considered as the same as the running time of deconvolution step which can be also found in Table 2. In other words, the final performance depends on *Deconvolution* step and the overall speedup factor is 3.74, which is very close to 3.75 from *Deconvolution*.

Lorenz et al. [16] did experiments on deconvolution using local AIFs. They did performance experiments on a small data set size, which was 128 × 128 voxels per slice, 11 slices and the number of time intervals was 44, one of the CT image sizes. The overall running time to finish their deconvolution is still 6 min (the same as in our experiments) with a speedup factor of 5.56. This is reduced to 1 min and 5 s after applying parallelism. However, in MR images, the data size has increased to 128 × 128 voxels per slice, 22 slices and the number of time intervals is now 80, approximately four times as much data. It costs about 35 min in our serial implementation.^5^

The use of four threads in OpenMP parallelization provides speedup factors of 2.21 and 2.26 for the MR image size data and CT image size data, respectively. Parallelization using MPI leads to a better performance compared with OpenMP, which results in speedup factors of 3.42 and 3.84, respectively.^6^ For MR image size data, the GPGPU approach takes 59% of the time of OpenMP approach and 91% of the time of MPI approach. For CT image size data, our GPGPU approach has more than double the performance of the OpenMP method and has 1.45 times performance of the MPI method. Thus, for both of the MR image size data and CT image size data, our GPGPU parallel implementation shows a better performance than parallelizing over four CPUs.

### 4.4 GPGPU parameters

**Fig. 5:**
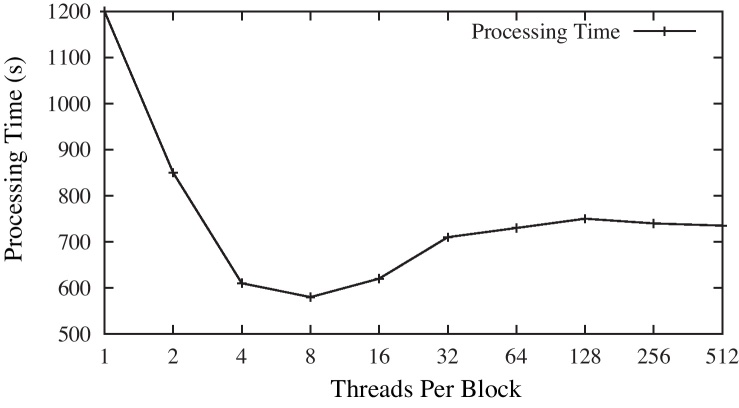
Threads per block. This figure shows the relationship between the parameter *Threads Per Block* and processing time. Note that the *X*-axis is in logarithmic (base 2) scale.

Fig. 5 shows that performance changes with the number of threads per block. According to the design of CUDA, all threads of a block should assigned to a same processor core. As the total number of threads is stationary, if the number of threads per block is too small, it will also lead to a large number of blocks and therefore lead to extra scheduling overhead. On the other hand, the tasks for each thread are very heavy, the best performance is not achieved at 128 or 256 threads per block but with a smaller number. This is because each thread is heavy, if there are too many threads in one block, the performance is restricted to the limited memory resources of a processor core. As a result, increasing the number of threads per block further cannot gain more speed up. As shown in Fig. 5, we achieve the peak performance when setting the number of threads per block to eight.

Furthermore, since the workload of each thread is very small compared to the whole task, load balance is not an important performance factor when changing the number of threads per block parameter.

## 5 Conclusion

In this paper, we introduced an implementation of perfusion-imaging analysis which provides considerable speed improvement and equivalent quality of results compared with current serial implementations. We have analyzed every individual step in the perfusion imaging processing. The *Deconvolution* step is the bottleneck for perfusion-imaging analysis, although the speedup factor is more than a hundred for both the *Data reorganization* and *Denoising* steps, the overall performance speedup factor is limited by this bottleneck. The overall processing time is reduced from 6 min to 65 s for CT images, from 35 min to less than 10 min for MR images. The performance speedup factors are 5.56 and 3.75, for CT and MR images respectively. Meanwhile, the quality of serial and parallel output images is unchanged. The speedup also depends on the CUDA configuration parameters which determine how tasks are assigned to GPU cores. In clinical diagnosis, time is vitally important especially for acute stroke cases, the earlier we deliver the result for diagnosis, the less damage will be caused by stokes and the higher the possibility that treatment will be effective. Therefore, performance is as important as accuracy in perfusion imaging, and our implementation can be used to help clinical diagnosis.

Our implementation using GPGPU can significantly reduced analysis processing time based on local AIFs, which makes it possible to use local AIFs in clinical diagnosis. Our experiment also shows that GPGPU implementation is superior to four cores CPU implementations. In conclusion, using GPGPU has several advantages for perfusion-imaging analysis.

Furthermore, with the improvement of CT and MR imaging, the size of input images are likely to expand. Thus the processing time of perfusion weighted image analysis will tend to rise which will definitely increase the demand for speedup by exploiting parallel hardware.

## Figures and Tables

**Table 1:** Computation time for SVD (in seconds).

Matrix size	MATLAB	MKL	GPGPU
64 × 64	0.01	0.003	0.054
128 × 128	0.03	0.014	0.077
256 × 256	0.210	0.082	0.265
1K × 1K	72	11.255	3.725
2K × 2K	758.6	114.625	19.6
4K × 4K	6780	898.23	133.68

The column on the left indicates the size of each matrices; the second column is the result for MATLAB and third one is the result for Intel math kernel library LAPACK. The column on the right are the results of Lahabar's work that using GPGPU to decomposition.

**Table 2:** Performance of each step.

Step	Serial running time	Parallel running time (s)	Speedup factor
Brain data load	0.10	0.10	–
Data copying (CPU to GPU)	Not applied	0.17	–
Data reorganization	1.1	0.01	110
Reorganization and denoising	4.3	0.01	430
Deconvolution	2108	564	3.74
Data copying (GPU to CPU)	Not applied	0.01	–
Draw parametric maps	0.20	0.20	–
Overall	2114	564	3.75

This table indicates the processing time of both serial and parallel algorithms for each individual step. Because of *Brain data load* and *draw parametric maps* steps are the same in both serial and parallel algorithm and *Data copying* steps only happen in parallel algorithm, speedup factors are not calculated for these steps.

**Table 3:** Overall performance.

Data size (*Dim*1 × *Dim*2 × *Dim*3 × *time*)	Serial running time (s)	GPGPU running time (s)	OpenMP running time	MPI running time (s)
128 × 128 × 22 × 80	2114	564	956	619
(MR image size)		Speedup factor = 3.75	Speedup factor = 2.21	Speedup factor = 3.42
128 × 128 × 11 × 44	360	65	159	94
(CT image size)		Speedup factor = 5.56	Speedup factor = 2.26	Speedup factor = 3.84

This table indicates the overall running time and speedup factor for all of the serial and parallel implementations.

## Footnotes

1 4 × 80^2^ × 8 bytes = 204, 800 bytes.

2 128 × 128 × 22 × 200 KB = 68.75 GB.

3 128 × 128 × 22 × 80 × 2 Byte (*short* data type) = 55 MB.

4 3 × 128 × 128 × 22 × 1 Byte = 1.03125 MB.

5 It will cost around 40 min using Lorenz's methods by computational complexity estimation.

6 Considering that the number of CPU cores in the experiments is four, the theoretical upper boundary of performance improvement is 4.
